# Transcriptome-wide analysis of differentially expressed chemokine receptors, SNPs, and SSRs in the age-related macular degeneration

**DOI:** 10.1186/s40246-019-0199-1

**Published:** 2019-03-20

**Authors:** Madhu Sudhana Saddala, Anton Lennikov, Anthony Mukwaya, Lijuan Fan, Zhengmao Hu, Hu Huang

**Affiliations:** 1 Mason Eye Institute, University of Missouri, Columbia, MO 65212 USA; 2 Wilmer Eye Institute, Johns Hopkins University, Baltimore, MD 21287 USA; 3 Department of Ophthalmology, Faculty of Health Sciences, Institute for Clinical and Experimental Medicine, Linköping University, SE-581 83 Linköping, Sweden; 4 Center for Medical Genetics and Hunan Key Laboratory of Medical Genetics, School of Life Sciences, Central South University, Changsha, China; 5 Department of Ophthalmology, School of Medicine, University of Missouri-Columbia, 1 Hospital Drive, MA102C, Columbia, MO 65212 USA

**Keywords:** AMD, RNA-Seq, Gene ontology, Human eye, Chemokine receptor

## Abstract

**Background:**

Age-related macular degeneration (AMD) is the most common, progressive, and polygenic cause of irreversible visual impairment in the world. The molecular pathogenesis of the primary events of AMD is poorly understood. We have investigated a transcriptome-wide analysis of differential gene expression, single-nucleotide polymorphisms (SNPs), indels, and simple sequence repeats (SSRs) in datasets of the human peripheral retina and RPE-choroid-sclera control and AMD.

**Methods and results:**

Adaptors and unbiased components were removed and checked to ensure the quality of the data sets. Molecular function, biological process, cellular component, and pathway analyses were performed on differentially expressed genes. Analysis of the gene expression datasets identified 5011 upregulated genes, 11,800 downregulated genes, 42,016 SNPs, 1141 indels, and 6668 SRRs between healthy controls and AMD donor material. Enrichment categories for gene ontology included chemokine activity, cytokine activity, cytokine receptor binding, immune system process, and signal transduction respectively. A functional pathways analysis identified that chemokine receptors bind chemokines, complement cascade genes, and create cytokine signaling in immune system pathway genes (*p* value < 0.001). Finally, allele-specific expression was found to be significant for Chemokine (C-C motif) ligand (CCL) 2, 3, 4, 13, 19, 21; C-C chemokine receptor (CCR) 1, 5; chemokine (C-X-C motif) ligand (CXCL) 9, 10, 16; C-X-C chemokine receptor type (CXCR) 6; as well as atypical chemokine receptor (*ACKR*) 3,4 and pro-platelet basic protein (PPBP).

**Conclusions:**

Our results improve our overall understanding of the chemokine receptors’ signaling pathway in AMD conditions, which may lead to potential new diagnostic and therapeutic targets.

**Electronic supplementary material:**

The online version of this article (10.1186/s40246-019-0199-1) contains supplementary material, which is available to authorized users.

## Background

Age-related macular degeneration (AMD) is the most common, progressive, and polygenic disease responsible for visual impairment in individuals over the age of 60 [1]. The number of individuals with AMD has steadily increased, and it is estimated to reach 196 million people affected by 2020, given the increasing longevity of the worldwide population [2]. Because AMD is a progressive degenerative disease, having the intermediate stage puts one at risk for the more advanced forms of AMD. Clinically, two forms of advanced AMD are recognized: geographic atrophy and neovascular AMD. Geographic atrophy is characterized by the loss of an area of the RPE and choroid that results in the gradual decline in the number of photoreceptors. Neovascular or “wet” form of AMD is characterized by the growth of abnormal new blood vessels from the choroid into sub-RPE and subretinal regions. These poorly developed neovessels cause hemorrhage and exudation of fluid in the macular region interfering with the function of the retina leading to loss of vision. Even though treatments aimed at inhibiting blood vessel growth can effectively slow the progression of “wet” AMD, no useful treatments exist for the atrophic (“dry”) form of the disease, which accounts for the majority of all AMD cases [3]. Several biological processes have been implicated in the pathogenesis of AMD, including complement activation [4], inflammation [5], and oxidative stress [6]. Studies of gene expression regarding the AMD phenotype are becoming increasingly important in assessing the relevance and possible functions of AMD risk loci and in moving beyond genetic association to uncover the specific pathways involved in the development and progression of the disease [7, 8]. Many of the studies to date investigating AMD gene association are based on genome-wide association (GWA) [9] such as association with HtrA serine peptidase 1 (HTRA1)/age-related maculopathy susceptibility 2 (ARMS2) and CFH [10, 11]. However, most of the variants investigated so far are tag single-nucleotide polymorphisms (SNPs) or noncoding SNPs within large intergenic regions, thus making it difficult to ascertain the function of disease-associated variants. Coding variants can cause truncated transcripts that can affect protein synthesis, and function or stability. Here, RNA-Seq allows for a global analysis of the transcriptome of the affected tissue in an unbiased manner with the capacity to measure the expression of individual genes [6]. Moreover, RNA-Seq analysis can also assess the different isoforms of each gene, alternative splice events, novel, and rare transcripts, and identify the role of ncRNAs. RNA-Seq also has a lower frequency of false-positive rates and a higher reproducibility [12, 13].

Understanding which genes are perturbed in the pathophysiology of AMD could pave the way for the identification of biomarkers as potential predictors of disease onset and can be validated as potential therapeutical targets. Also, the identification of potential therapies may be facilitated by high-throughput systems using biological analyses, particularly at the transcriptome levels.

Global gene expression assays, however, only provide information about the transcriptome profile at the time of sampling which in turn can be affected by the rate of translation, and by the rate of protein turnover [14], thus, all genomics, proteomics, and transcriptome analysis methodologies are required for a complete understanding of gene and protein interaction involved in the AMD pathophysiology.

Here, publicly available RNA-Seq datasets representing total mRNA harvested from human ocular tissues from healthy and AMD donors were analyzed using unique analysis pipelines. The dataset was analyzed for differentially expressed genes (DEGs) and using these DEGs, pathway enrichment analysis and functional annotation analyses were performed. From the results, we identify the most dysregulated pathways such as chemokine signaling pathway, complement cascade pathway, and cytokine signaling, and biological processes, such as cell communication, cell surface receptor signaling pathway, signal transduction, biological adhesion, and numerous chemokine receptors, which may be significant players in the pathophysiology of AMD.

## Methods

### Data source, quality, and preprocessing

The human healthy and AMD condition in the retina and RPE-choroid-scleral (RCS) RNA-Seq raw paired-end datasets obtained from the Sequence Read Archive (SRA) (https://www.ncbi.nlm.nih.gov/sra/?term=SRP107937) from the National Centre for Biotechnology Information (NCBI). Kim et al. produced the original dataset. [15] Eight AMD and healthy Caucasian close age-matched donor eyes datasets were used, PMI < 6 h. The mean age for AMD donors was 84.1 ± 6.9 years, and the healthy donor was 85 ± 3.1 years. Donors in AMD group were diagnosed histologically with early-stage non-exudative age-related maculopathy (three donors, six datasets); late stage non-exudative age-related maculopathy (four donors, eight datasets); and RPE cell epithelial dystrophy (one donor, two datasets). Donors in the healthy control group were selected based on the absence of ocular pathology. Equal weight was assigned to retinal and RPE/choroid material with a dataset from the same donor. Detailed information of datasets is presented in Table 1.

**Table 1:** Detailed information of age macular degeneration (AMD) datasets with experiment, runs, sex, age, donor id, eye histological phenotype, and tissue [15]

Experiment	Run	Sex	Age	Donor Id	Eye histological phenotype	Tissue
SRX2848433	SRR5591599	Male	85	Normal_1	Normal	Peripheral retina
SRX2848434	SRR5591600	Male	85	Normal_1	Normal	Peripheral RPE-choroid-sclera
SRX2848435	SRR5591601	Male	84	Normal_2	Normal	Peripheral retina
SRX2848436	SRR5591602	Female	92	Normal_3	Normal	Peripheral retina
SRX2848437	SRR5591603	Female	92	Normal_3	Normal	Peripheral RPE-choroid-sclera
SRX2848438	SRR5591604	Female	86	Normal_4	Normal	Peripheral retina
SRX2848439	SRR5591605	Female	86	Normal_4	Normal	Peripheral RPE-choroid-sclera
SRX2848440	SRR5591606	Male	83	Normal_5	Normal	Peripheral retina
SRX2848441	SRR5591607	Male	83	Normal_5	Normal	Peripheral RPE-choroid-sclera
SRX2848442	SRR5591608	Male	83	Normal_6	Normal	Peripheral retina
SRX2848443	SRR5591609	Male	83	Normal_6	Normal	Peripheral RPE-choroid-sclera
SRX2848444	SRR5591610	Male	84	Normal_7	Normal	Peripheral retina
SRX2848445	SRR5591611	Male	84	Normal_7	Normal	Peripheral RPE-choroid-sclera
SRX2848446	SRR5591612	Female	83	Normal_8	Normal	Peripheral retina
SRX2848447	SRR5591613	Female	83	Normal_8	Normal	Peripheral RPE-choroid-sclera
SRX2848448	SRR5591614	Male	69	AMD_1	Early age-related maculopathy	Peripheral retina
SRX2848449	SRR5591615	Male	69	AMD_1	Early age-related maculopathy	Peripheral RPE-choroid-sclera
SRX2848450	SRR5591616	Female	85	AMD_2	Early age-related maculopathy	Peripheral retina
SRX2848451	SRR5591617	Female	85	AMD_2	Early age-related maculopathy	Peripheral RPE-choroid-sclera
SRX2848452	SRR5591618	Male	95	AMD_3	Late non-exudative age-related maculopathy	Peripheral retina
SRX2848453	SRR5591619	Male	95	AMD_3	Late non-exudative age-related maculopathy	Peripheral RPE-choroid-sclera
SRX2848454	SRR5591620	Male	87	AMD_4	Late exudative age-related maculopathy	Peripheral retina
SRX2848455	SRR5591621	Male	87	AMD_4	Late exudative age-related maculopathy	Peripheral RPE-choroid-sclera
SRX2848456	SRR5591622	Female	83	AMD_5	Late non-exudative age-related maculopathy	Peripheral retina
SRX2848457	SRR5591623	Female	83	AMD_5	Late non-exudative age-related maculopathy	Peripheral RPE-choroid-sclera
SRX2848458	SRR5591624	Female	86	AMD_6	Other (RPEcell epithelial dystrophy (suspected))	Peripheral retina
SRX2848459	SRR5591625	Female	86	AMD_6	Other (RPEcell epithelial dystrophy (suspected))	Peripheral RPE-choroid-sclera
SRX2848460	SRR5591626	Female	86	AMD_7	Late non-exudative age-related maculopathy	Peripheral retina
SRX2848461	SRR5591627	Female	86	AMD_7	Late non-exudative age-related maculopathy	Peripheral RPE-choroid-sclera
SRX2848462	SRR5591628	Female	86	AMD_8	Early age-related maculopathy	Peripheral retina
SRX2848463	SRR5591629	Female	86	AMD_8	Early age-related maculopathy	Peripheral RPE-choroid-sclera

**Fig. 1:**
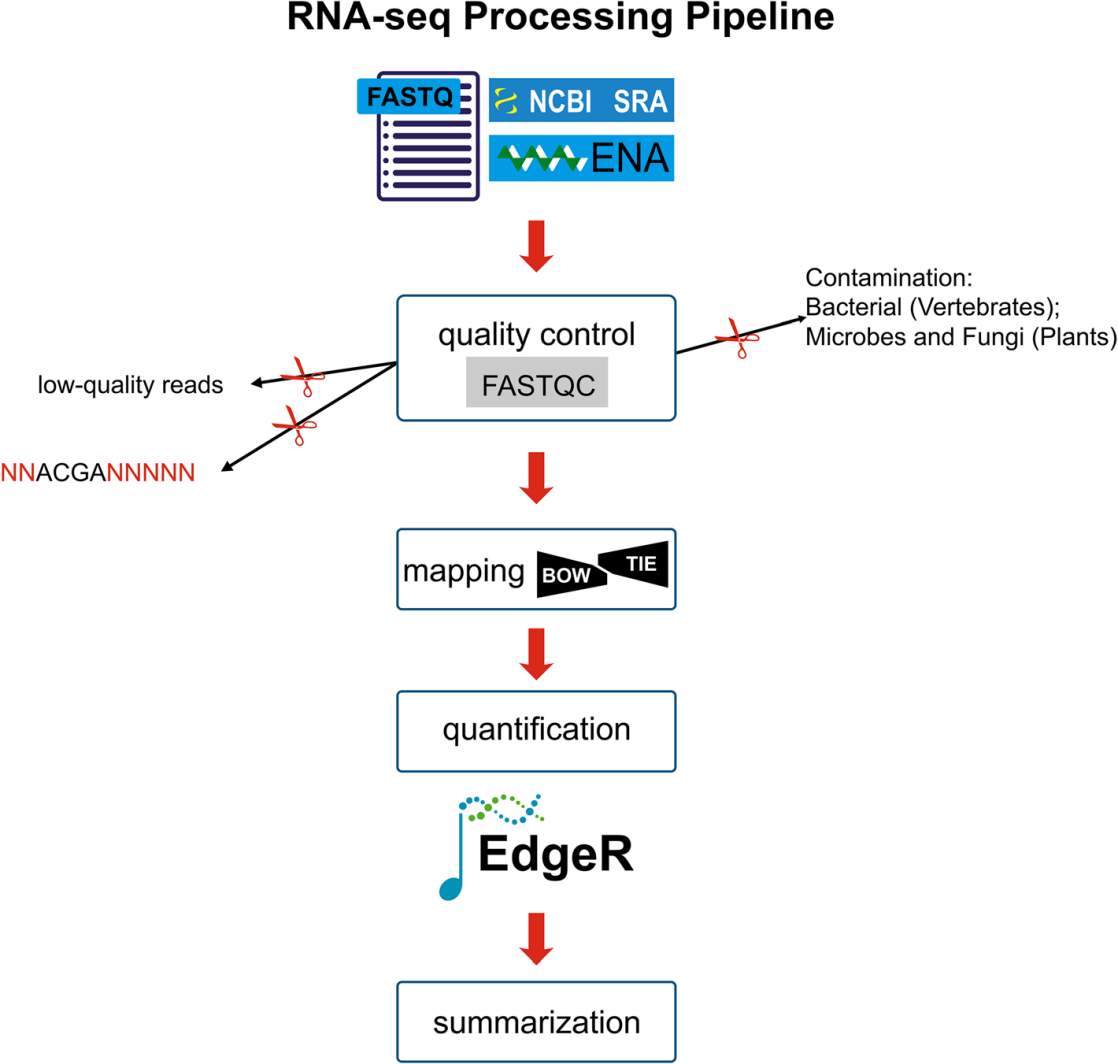
The basic workflow of RNA-Seq analysis. The human data sets collected, quality checked, reference genome mapped, and gene expression quantification. Gene ontology, functional pathways, gene network analysis, and summarization

Kim et al. who created and deposited the datasets focused on the significant differential expression of noncoding RNA and antisense transcripts in AMD [15], whereas our current work focuses on chemokines receptors and their pathways, in the pathophysiology of AMD, using bioinformatics analysis pipeline (Fig. 1), using the SRA Toolkit (https://www.ncbi.nlm.nih.gov/sra/docs/toolkitsoft/). The SRA files were converted to a fastq format into forward and reverse separate files using the SRA Toolkit split function. These raw reads were used for visualization of the reads’ quality before and after preprocessing by using FastQC software (https://www.bioinformatics.babraham.ac.uk/projects/fastqc/). The process to remove adapters and ambiguous quality reads were done using the Trimmomatic-0.36 tool, here trimming of bases from 3′ and 5′ ends, maintaining the Phred-score at ≤ 30.

### Reference-based assembly

The human genome was aquired from the NCBI genome (https://www.ncbi.nlm.nih.gov/genome/?term=human) for reference-based assembly. All the datasets were assembled separately with reference genomes using Bowtie software [16]. Initially, Bowtie indexed the genome with a Burrows-Wheeler index to keep its memory footprint small. Finally, the RNA-Seq by expectation-maximization (RSEM) tool extracted the reference transcripts from a genome with gene annotations in a GTF file. All the annotation files were merged to obtain a single assembly file [17]. We obtained good assembly results with the human dataset when validating the assembly read remapping that was conducted using bowtie2 for each data set, creating the bowtie2 index [16]. CAP3 assembler was used to keep only the longest isoform for each gene to reduce redundancy [18].

### Identification of DEGs

Trinity software supports the use of Bioconductor tool such as EdgeR to analyze differential expression analysis in the assembled transcriptome. Finally, the both healthy controls and AMD comparison transcript counts (matrix file) were used for differential gene expression using edgeR (Empirical analysis of digital gene expression in R) package of Bioconductor with primary parameters such as false discovery rate (FDR), log fold-change (logFC), log counts per million (logCPM), and *p* value [19, 20]. Unigenes with adjusted *q* value less than 0.001 (*p* < 0.001) and the fold changes more than 4 (logFC > 4) were considered as significantly differentially expressed unigenes.

### Functional annotation

Gene ontology (GO) is an internationally standardized gene functional classification system that offers a dynamically updated controlled vocabulary and a strictly defined concept to comprehensively describe the properties of genes and their products in any organism. The identified DEGs were subjected to further functional analysis using the GO enrichment analysis [21]. DEGs were assigned to their biologically relevant pathways using the KEGG pathway database through the KEGG Automated Annotation Server (KAAS) [22]. Hypergeometric test using the GO terms and the KEGG pathway (Kyoto Encyclopedia of Genes and Genomes, http://www.kegg.jp/) terms were performed, and the analysis of significant GO terms or KEGG pathway terms were identified with a *p* value < 0.05.

### Identification of SNPs and indels

The good quality clean reads from each sample were aligned individually to reference the transcriptome using a Burrows-Wheeler Aligner (BWA). The read count profile from the output file (.sam file) of the BWA alignment was generated by using samtools. For SNP identification, the sorted alignment files from each sample were used to produce a bcf file using samtools [23]. The bcf tool was used to filter the SNPs at a *p* value < 0.001. The potential SNPs were identified using read depth (*d*) ≥ 10, quality depth (*Q*) ≥ 30, minimum root mean square mapping quality (MQ) ≥ 40, and flanking sequence length (*l*) = 50.

### Identification of simple sequence repeats

The identification of simple sequence repeats (SSRs) in the unigenes of healthy and AMD human datasets was predicted using the Perl script MISA (MIcroSAtellite; http://pgrc.ipk-gatersleben.de/misa) tool [24]. Dinucleotide repeats of more than six times and tri, tetra, penta, and hexanucleotide repeats of more than five times were considered the search criteria for SSRs.

### Gene network analysis

We also performed the functional enrichment and interaction network analysis using the STRING 10.5 database [25] on using the DEGs as the input files. STRING tool classified the DEGs according to the GO categories, such as biological processes (BP), molecular function (MF), cellular components (CC), and Kyoto Encyclopedia of Genes and Genomes database (KEGG) pathways.

## Results

### Quality and pre-processing of datasets

The RNA-Seq paired-end human healthy and AMD data (SRP107937) were acquired from the National Centre for Biotechnology Information-Sequence Read Archive (NCBI-SRA) using the SRA Toolkit (https://www.ncbi.nlm.nih.gov/sra/docs/toolkitsoft/) with a prefetch function, save for one file (SRR5591614). The paired 30 SRA files were converted into fastq files (60 files) with fastq-dump and split-files functions.

Initially, visualization of the quality of all datasets before and after trimming the adaptors and going through the pre-processing steps was performed using a FastQC tool (https://www.bioinformatics.babraham.ac.uk/projects/fastqc/). Finally, low-quality reads were removed by trimming the bases from 3′ and 5′ end and maintaining the Phred-score ≤ 30 using the Trimmomatic-0.36 tool [26]. After cleaning and trimming of low-quality reads and adaptor removal, more than 97% good quality reads in each stage were retained. The human healthy and AMD of paired-end raw data before and after elimination of adapters and retained reads percentages were tabulated (Additional file 1: Table S1). These cleaned reads were used for further transcriptome assembly analysis.

### Reference-based assembly

The healthy and AMD datasets were assembled separately with a reference human genome using Bowtie software [16]. The RSEM (RNA-Seq by expectation-maximization) tool can extract reference transcripts from a genome with gene annotations in a GTF file. From this extraction, 93% and 95% of the reads from healthy and AMD, respectively, were successfully mapped on a reference transcriptome. All the annotation files were merged to obtain a single assembly file [17]. The assembled files were used for removing redundant sequences with CAP3 assembler software [18]. However, the CAP3 assembler generated 139,575 contigs and 1,610,520 singlets. Both the contig and singlet files were combined, and sequences below 300 in length were removed. The final output file was considered the reference genome for further analysis. All the samples were given equal weight within a control dataset and a disease dataset. Therefore, all samples (*n* = 8) control (neural retina and RPE/choroid) vs. all AMD comparison (neural retina and RPE/choroid) were used for the analysis.

### Differential gene expression analysis

**Fig. 2:**
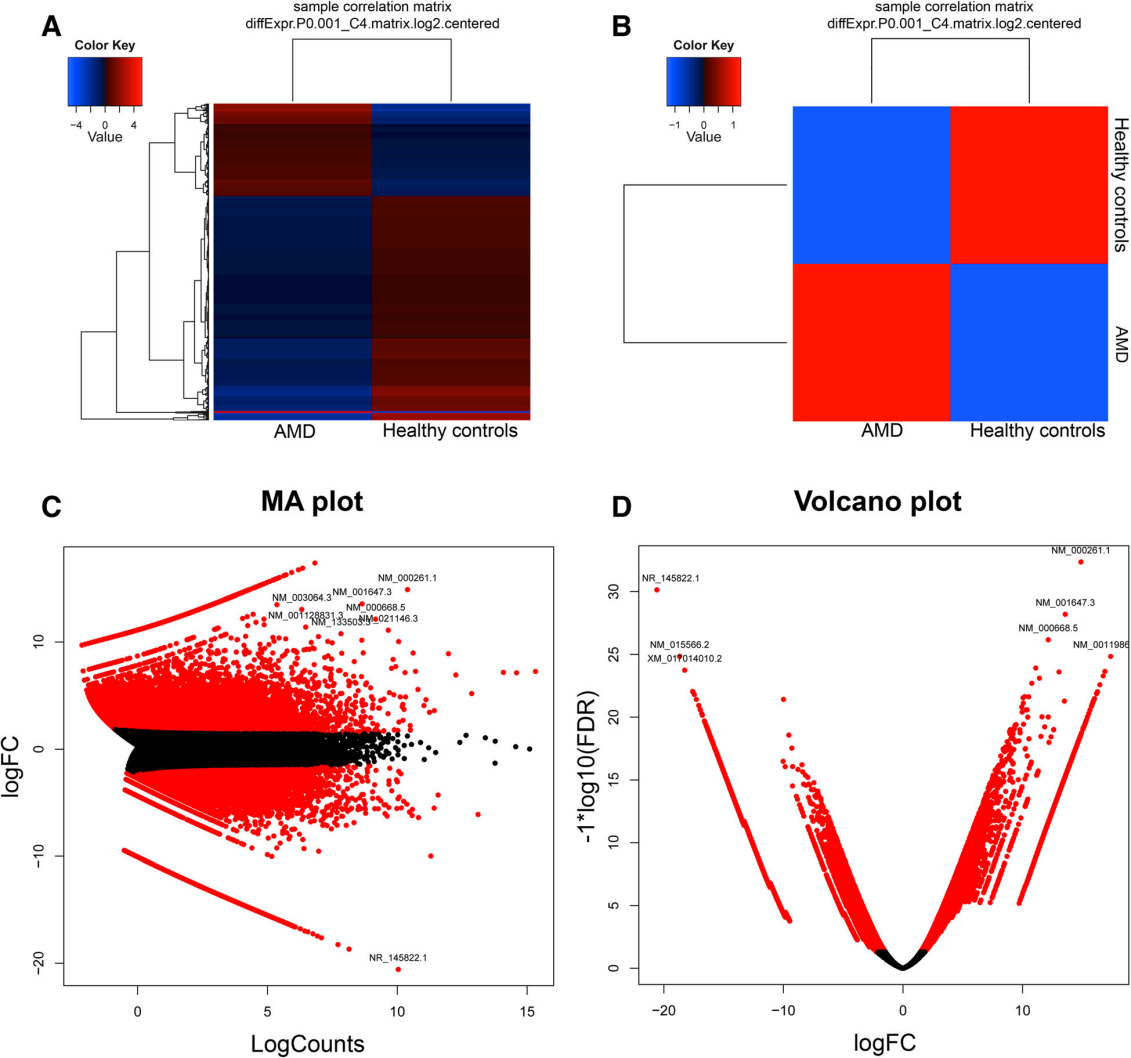
Graphical representation of differentially expressed genes. **a** Significantly up- and downregulated genes were represented as a heat map (red color shows upregulated and blue color shows downregulated genes. **b** Significantly up- and the downregulated gene was represented as a matrix correlation plot **c** MA plot represented by significantly up- and downregulated gene based on the logFC and logCounts. **d** Volcano plot represented by significantly up- and downregulated gene based on the logFC and log10 (FDR)

RSEM software was used to calculate the expression values in the form of fragments per kilobase of exon per million mapped reads (FRKM). Finally, both the healthy and AMD comparison transcript counts (matrix file) were used for differential gene expression using edgeR (empirical analysis of digital gene expression in R) package of Bioconductor with primary parameters such as the false discovery rate (FDR), log fold change (logFC), log counts per million (logCPM), and *p* value Unigenes with a *p* value less than 0.001 (*p* < 0.001) and a fold change of more than 4 (logFC >  4) were considered significantly differentially expressed genes. Principle component analysis (PCA) was performed for all the samples in both groups, and the results demonstrated that samples within the AMD group were separate from those in the control group. Furthermore, PCA screen plot confirmed that principle component 1 (PC1) and 2 (PC2) accounted for 98% of the total variation in gene expression. To further investigate the two groups, we performed hierarchical clustering of all DEGs. Extract clusters of transcripts with similar expression profiles by cutting the transcript cluster dendrogram at a given percent of its height (60%), creating individual transcript clusters and summarizes expression values for each cluster according to individual charts (Additional file 1: Figure S2). We discovered 5011 upregulated and 11,800 downregulated DEGs among the 51,532 totals, in AMD conditions relative to healthy controls datasets. The hierarchical clustering heatmap, correlation matrix plot, MA plot, and volcano plots were generated to represent the up- and downregulated genes (Fig. 2).

### Functional annotation

In the current study, functional assignment and categorization for the identified DEGs were carried out using a GO analysis by the GO enrichment analysis [21]. All the up- and downregulated genes were uploaded to the GO enrichment analysis tool with the complete human genome as the background. The MFs, BPs, CC protein classes, and pathways were predicted in the significantly enriched GO terms.

**Fig. 3:**
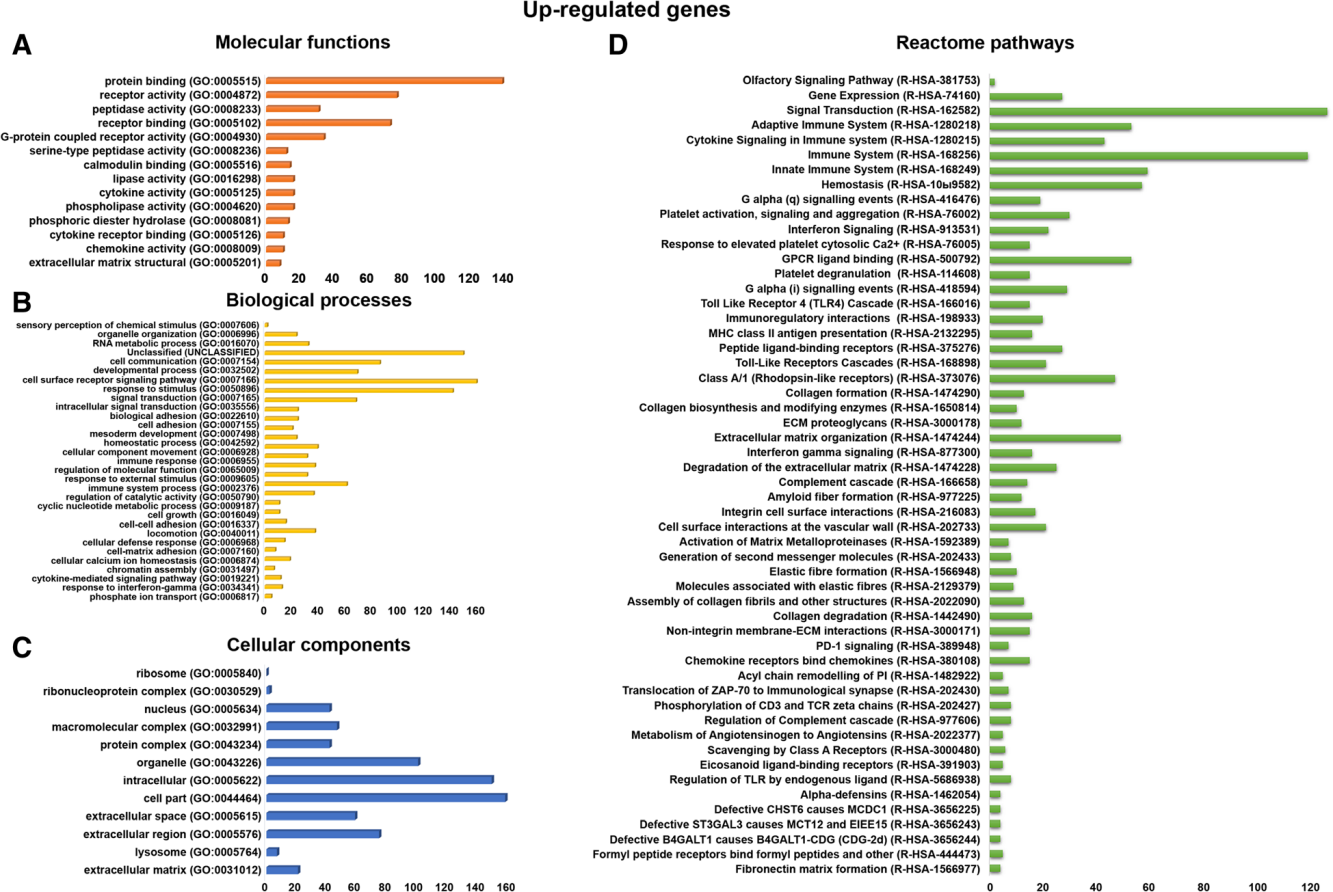
Gene ontology and Reactome pathways of up-regulated genes. **a** The upregulated genes are involved in different molecular functions. **b** The upregulated genes are involved in a different biological process. **c** The upregulated genes are involved in different cellular components functions. **d** The upregulated genes are involved in various Reactome biological pathways

The upregulated genes were involved in various molecular functions, such as protein binding (139 genes), receptor activity (77 genes), receptor binding (73 genes), G protein-coupled receptor activity (34 genes), cytokine activity (16 genes), cytokine receptor binding (10 genes), chemokine activity (10 genes), and extracellular matrix structural constituent (8 genes), respectively (Fig. 3a). The upregulated genes were involved in various biological processes, such as RNA metabolic process (33 genes), cell communication (150 genes), cell surface receptor signaling pathway (70 genes), signal transduction (142 genes), biological adhesion (25 genes), cellular component movement (40 genes), regulation of molecular function (38 genes), immune system process (62 genes), cellular calcium ion homeostasis (19 genes), and cytokine-mediated signaling pathway (12 genes) (Fig. 3b). The upregulated genes were involved in various cellular components, such as nucleus (42 genes), macromolecular complex (47 genes), extracellular matrix (21 genes), protein complex (42 genes), organelle (101 genes), intracellular (150 genes), cell part (159 genes), extracellular space (59 genes), extracellular region (75 genes), and extracellular matrix (27 genes), respectively (Fig. 3c).

**Fig. 4:**
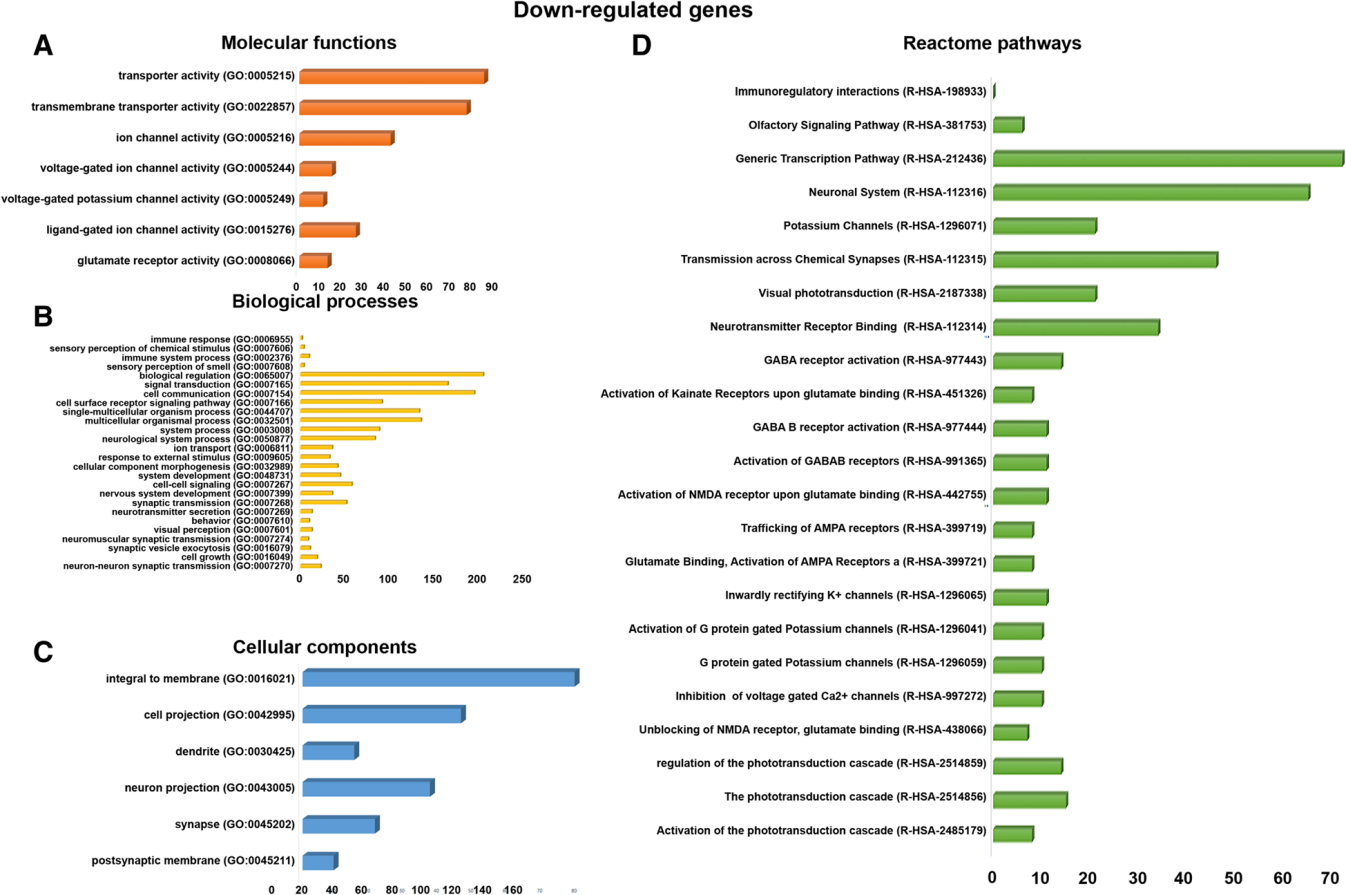
Gene ontology and Reactome pathways of downregulated genes. **a** The downregulated genes are involved in different molecular functions. **b** The downregulated genes are involved in a different biological process. **c** The downregulated genes are involved in different cellular components functions. **d** The downregulated genes are involved in various Reactome biological pathways

The downregulated genes were involved in various molecular functions, such as transporter activity (85 genes), transmembrane transporter activity (77 genes), ion channel activity (42 genes), ligand-gated ion channel activity (26 genes), and glutamate receptor activity (13 genes), respectively (Fig. 4a). The downregulated genes were involved in various biological processes, such as immune system process (10 genes), signal transduction (166 genes), cell surface receptor signaling pathway (92 genes), multicellular organismal process (136 genes), neurological system process (84 genes), nervous system development (36 genes), neurotransmitter secretion (13 genes), visual perception (13 genes), and neuron-neuron synaptic transmission (23 genes), respectively (Fig. 4b). The downregulated genes were involved in various cellular components, such as integral to membrane (79 genes), cell projection (46 genes), dendrite (15 genes), neuron projection (37 genes), synapse (21 genes), and postsynaptic membrane (9 genes), respectively (Fig. 4c).

### Pathways analysis

We identified the upregulated genes involved in different reactome pathways, such as extracellular matrix organization (49 genes), peptide ligand-binding receptors (27 genes), class A/1 (rhodopsin-like receptors) (47 genes), GPCR ligand binding (53 genes), signal transduction (126 genes), innate immune system (59 genes), immune system (119 genes), regulation of TLR by endogenous ligand (8 genes), toll-like receptors cascades (21 genes), metabolism of angiotensinogen to angiotensin (5 genes), complement cascade (14 genes), adaptive immune system (53 genes), chemokine receptors bind chemokines (15 genes), collagen degradation (16 genes), degradation of the extracellular matrix (25 genes), assembly of collagen fibrils and other multimeric structures (13 genes), collagen formation (13 genes), activation of matrix metalloproteinases (7 genes), cell surface interactions at the vascular wall (21 genes), hemostasis (57 genes), integrin cell surface interactions (17 genes), amyloid fiber formation (12 genes), interferon gamma signaling (16 genes), cytokine signaling in immune system pathway (43 genes), ECM proteoglycans (12 genes), MHC class II antigen presentation (16 genes), immunoregulatory interactions between a lymphoid and a non-lymphoid cell (20 genes), toll-like receptor 4 (TLR4) cascade (15 genes), G alpha (i) signaling events (29 genes), platelet activation, signaling, and aggregation (30 genes), G alpha (q) signaling events (19 genes), and unclassified pathways (388 genes) (Fig. 3d).

The downregulated genes were also involved in various Reactome pathways, such as phototransduction cascade (15 genes), visual phototransduction (21 genes), inactivation, recovery and regulation of the phototransduction cascade (14 genes), in neurotransmitter receptor binding and downstream transmission in the postsynaptic cell (34 genes), transmission across chemical synapses (46 genes), neuronal system (65 genes), GABA receptor activation (14 genes), activation of G protein-gated potassium channels (10 genes), inwardly rectifying K^+^ channels (11 genes), potassium channels (21 genes), trafficking of AMPA receptors (8 genes), glutamate binding, activation of AMPA receptors and synaptic plasticity (8 genes), activation of kainate receptors upon glutamate binding (8 genes), and generic transcription pathway (72 genes), respectively (Fig. 4d).

In the current study, we primarily focused on chemokine receptors binding in the chemokines pathway, complements to the cascade pathway, and cytokine signaling in immune system pathways. *CCL3*, *ACKR4*, *CCL19*, *CCL2*, *CXCL10*, *PPBP*, *CXCL9*, *CCL13*, *CCR1*, *CCL21*, *ACKR3*, *CXCL16*, *CCR5*, *CCL4*, and *CXCR6* are involved in the chemokine signaling pathway (Table 2). This pathway activates the JAK/Stat, Ras, ERK, and Akt pathways; NF-kappa B signaling pathway; and toll-like receptor signaling pathways. *C5AR1*, *CFH*, *C1R*, *C5AR2*, *CFD*, *C1QB*, *C1QC*, *CD59*, *CFP*, *C3AR1*, *C3*, *CR1*, *C1QA*, and *C1S* are involved in the complement cascade pathway (Additional file 1: Table S2). There are 43 genes involved in cytokine signaling in the immune system pathways (Additional file 1: Table S3).

**Table 2:** List of chemokine receptors bind chemokines pathway genes, UniProtKB, gene symbol, GenBank id, Gene name, logFC, *p* value KEGG pathways

UniProtKB	Gene symbol	GenBank ID	Gene name	logFC	*p* value	KEGG
P10147	CCL3	NM_002983	C-C motif chemokine 3	4.25663907	7.14E-07	hsa:6349
Q9NPB9	ACKR4	NM_016557	Atypical chemokine receptor 4	7.260759683	1.45E-14	hsa:51554
Q99731	CCL19	NM_006274	C-C motif chemokine 19	12.30108718	2.93E-14	hsa:6363
P13500	CCL2	NM_002982	C-C motif chemokine 2	5.52247071	6.79E-12	hsa:56477
P02778	CXCL10	NM_001565	C-X-C motif chemokine 10	6.770426736	4.33E-09	hsa:3627
P02775	PPBP	NM_002704	Platelet basic protein	6.083239276	4.56E-11	hsa:10895
Q07325	CXCL9	NM_002416	C-X-C motif chemokine 9	7.708086768	2.59E-10	hsa:4283
Q99616	CCL13	NM_005408	C-C motif chemokine 13	12.37011547	1.81E-14	hsa:6357
P32246	CCR1	NM_001295	C-C chemokine receptor type 1	6.996085978	4.47E-16	hsa:2826
O00585	CCL21	NM_002989	C-C motif chemokine 21	7.794499691	5.22E-08	hsa:6366
P25106	ACKR3	NM_020311	Atypical chemokine receptor 3	5.141169632	6.05E-11	hsa:57007
Q9H2A7	CXCL16	NM_001100812	C-X-C motif chemokine 16	5.167455173	1.45E-10	hsa:58191
P51681	CCR5	NM_001100168	C-C chemokine receptor type 5	5.636608022	7.70E-09	hsa:1234
P13236	CCL4	NM_002984	C-C motif chemokine 4	7.794499691	5.22E-08	hsa:6351
O00574	CXCR6	XM_005264809	C-X-C chemokine receptor type 6	4.131178122	3.85E-06	hsa:10663

### SNPs and indels identification

**Fig. 5:**
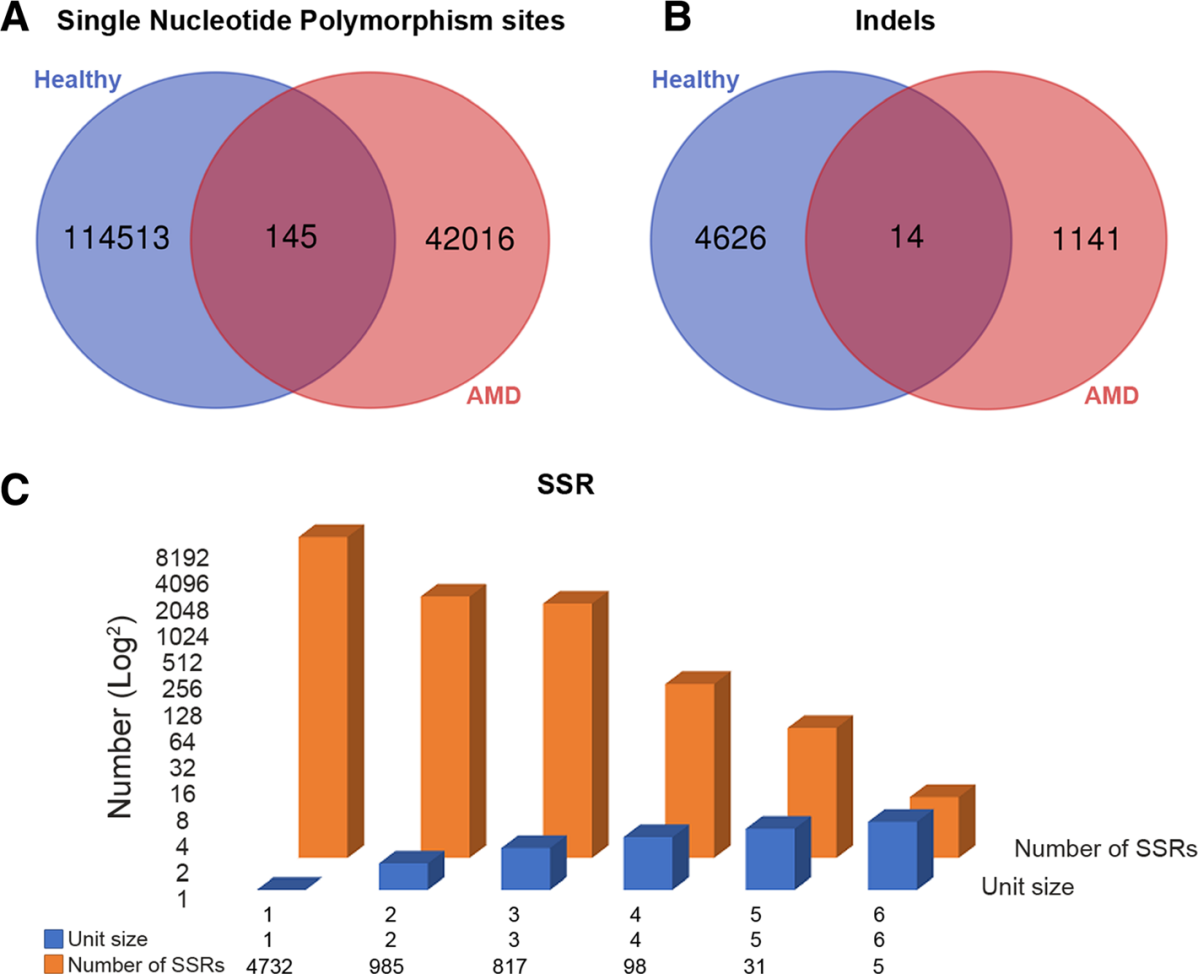
Comparisons of control vs. human AMD of the differentially expressed genes of SNPs and Indels were illustrated by Venn diagrams. **a** The Venn diagram is represented SNPs of differential expressed genes in control vs. human AMD. **b** The Venn diagram is represented Indels of differential expressed genes in control vs. human AMD. **c** The simple sequence repeats (SSRs) of differential expressed genes represented by bar diagram in human AMD

Since a significant number of SNPs and indels were initially mined, to narrow down the count, a quality value of 20 and read depth of 4 were applied as filtering criteria to identify significant SNPs and indels (Fig. 5a, b). We identified both the SNPs and indels in both the healthy and AMD human datasets; here, 114,513 SNPs were found in the healthy datasets, 42,016 SNPs were found in AMD datasets, and 145 SNPs were found in both the healthy and AMD datasets. For the indels, 4626 were found in healthy datasets, 1141 in AMD human datasets, and 14 in both data sets.

### SSRs markers identification

We performed SSRs identification in human healthy and AMD datasets using the MISA tool. The results showed the total number of identified SSRs (6668), number of SSR containing sequences (3340), number of sequences containing more than one SSR (1546), and number of SSRs present in compound formation (618). Among all the identified SSRs, A/T single-nucleotide SSRs (4653) have a higher distribution in unigenes. Figure 5c represents the information of mononucleotides (4732), dinucleotides (985), trinucleotides (817), tetranucleotides (98), pentanucleotides (31), and hexanucleotides (5) repeats.

### Gene network analysis

The up- and downregulated genes were used to construct gene-gene interactions using the STRING tool (https://string-db.org/), which also hid the disconnected nodes in the network. The results showed the analyzed number of nodes (581), expected number of edges (1113), a number of edges (1628), average node degree (5.6), average local clustering coefficient (0.374), and PPI enrichment *p* value < 1.0e-16. Overview of the nodes and total interactions tree is presented in Additional file 1: Figure S1.

**Fig. 6:**
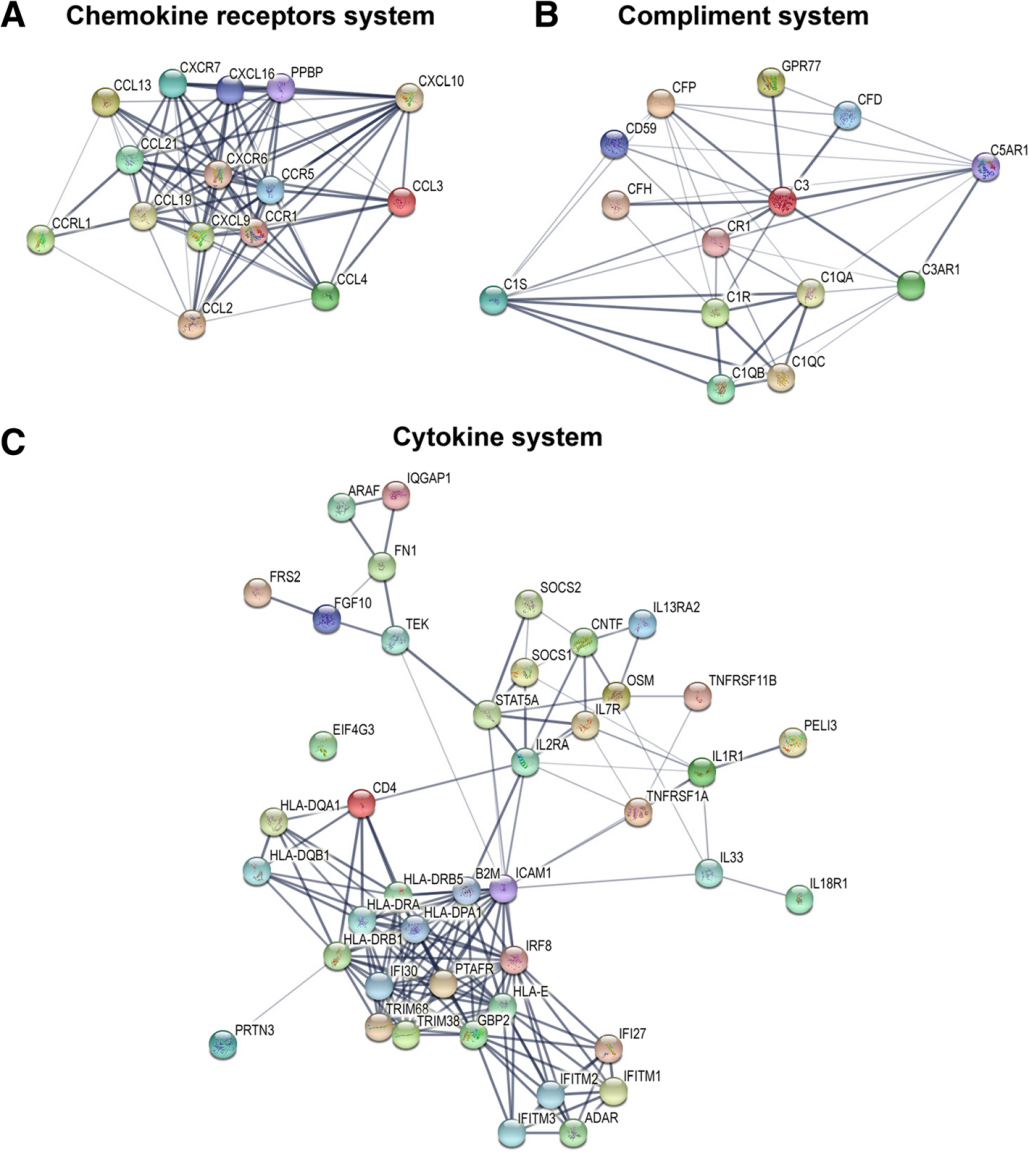
The functional enrichment and gene network analysis. **a** Subnetwork of chemokine signaling pathway genes. **b** Subnetwork complement and coagulation cascades pathway genes. **c** Subnetwork cytokine-cytokine receptor interactions pathway genes

Individual pathways nodes were isolated for the presentation of interactions. Figure 6 demonstrates that the chemokine signaling pathway (k04062) (Fig. 6a) has 15 nodes, 82 edges, 10.9 average node degree, 0.844 avg. local clustering coefficient, and 4 expected number of edges with a PPI enrichment *p* value < 1.0e-16. The complement pathway (k04610) (Fig. 6b) has 14 nodes, 44 edges, 6.29 average node degree, 0.695 avg. local clustering coefficient, and 1 expected number of edges with a PPI enrichment *p* value < 1.0e-16. The cytokine-cytokine receptor interactions pathway (k04060) (Fig. 6c) has 43 nodes, 164 edges, 7.63 average node degree, 0.69 avg. local clustering coefficient, and 14 expected number of edges with a PPI enrichment *p* value < 1.0e-16.

## Discussion

The present study elucidates the complete transcriptome sequencing (RNA-Seq) data analysis of healthy controls and AMD donor human eye tissues. Functional classification and pathway analysis revealed that the chemokine signaling pathway, complement cascade pathway, and cytokine signaling in immune system pathway genes and several other pathways are upregulated in ocular tissues. Chemokine receptors are small protein-coupled receptor (GPCR) superfamily and are activated on binding their cognate ligands of the chemokine family. Chemokine receptor binding initiates a cascade of intracellular events which are dissociation of the receptor-associated heterotrimeric G proteins into α and βγ subunits. Gα activates an enzyme known as phospholipase C (PLC) that is associated with the cell membrane. PLC cleaves phosphatidylinositol (4,5)-bisphosphate (PIP2) to form two second messenger molecules called inositol triphosphate (IP3) and diacylglycerol (DAG); DAG activates another enzyme called protein kinase C (PKC), and IP3 triggers the release of calcium from intracellular stores. These events promote many signaling cascades, affecting a cellular response. As per our results suggested that all the above-mentioned cascade signaling G protein pathway genes, phospholipase C gene, protein kinase C gene, ion channel genes: *GNA15*, *GNAO1*, *GNG3*, *GNG4*, *GNGT1*, *GNGT2*, *GRK4*, *GRK7*, *GPSM2*, *RGS3*, *PLCB2*, *PIK3CG*, *PTK2*, *PRKCZ*, *CACNA1A*, *CACNA1B*, *CACNA1E*, and *KCNJ9* are significantly upregulated in AMD conditions when compared with healthy controls.

Chemokines are also known to be upregulated in response to an infection and inflammation signals; they play critical roles in inflammatory and infectious diseases by regulating neutrophil, macrophage, and lymphocyte trafficking to the pathological sites [27, 28]. Leukocyte migration during conditions of tissue injury or infection is a multi-step process involving local upregulation of proinflammatory chemokine secretion in response to signaling molecules such as TNF-α and IFN-γ. Presentation of these chemokines on endothelial cell-surface glycosaminoglycans (GAGs), binding of chemokines to their cognate receptors on the leukocyte cell surface, activation of the leukocyte receptor, and migration of the leukocytes along the chemokine gradient within the extracellular matrix toward the site of chemokine release [29]. While in the eye, these processes are more complex due to the interplay of retinal microglia, RPE cells, and blood-retinal barrier (BRB) similar recruitment and activation of immune cells such as microglia is occurring.

Several chemokines implicated in AMD condition where *CCL3* is reported as a critical regulator of retinal inflammation, which is associated with the severity of retinal degeneration and *CCL3* is produced by subretinal microglia from the inner retina that can be a trigger for additional monocyte infiltration from the circulation via inner retinal blood vessels [30]. *CCL19* is known to be involved in immune-regulatory and inflammatory processes [31]. *CXCL7* is a protein belonging to the CXC-chemokine family and isoform of beta-thrombo-globulin or proplatelet basic protein (PPBP) [32]. Atypical chemokine receptor 4 (ACKR4) controls chemokine levels and localization via high-affinity chemokine binding and is uncoupled from classic ligand-driven signal transduction cascades, resulting in chemokine sequestration, degradation, or transcytosis [33]. *CCL2* is involved in the neuroinflammatory processes that take place in the various diseases of the central nervous system (CNS), which are characterized by neuronal degeneration [34]. Nawaz et al. reported that autocrine-autocrine *CCL2*, *CXCL4*, *CXCL9*, and *CXCL10* signals in retinal endothelial cells are enhanced in diabetic retinopathy [35]. *CCL13* is induced by the inflammatory cytokine interleukin-1 and TNF-α [36]. CXC-chemokine ligand 10 (*CXCL10*), which is a potent anti-angiogenic chemokine, is highly expressed in the retina of patients with AMD and laser-injured mice. Choroidal endothelial cells express CXC-chemokine receptor 3 (*CXCR3*), the *CXCL10* receptor, and genetic ablation of *CXCR3* exacerbates laser-induced choroidal neovascularization in mice [37]. Previous publications have indicated that some intraocular cytokine associated with inflammation decrease after anti-VEGF therapy in patients with AMD [38], and our results are consistent with these studies.

AMD is also associated with complementary activation or deregulation of the spontaneously initiated alternative complement pathway, leading to the local release of inflammatory activation products and local inflammation [39]. Multiple complement components, regulators, complement activation products, and inflammatory proteins have been identified to be upregulated in AMD conditions, including *C3*, *C3d*; the terminal components *C5*, *C6*, *C7*, *C8*, and *C9*; terminal complement regulators vitronectin and clusterin, apolipoproteins *apoA1*, *apoA4*, and *apoE*; as well as thrombospondin, serum amyloid A (*SAP-A*), and *SAP-P* [23, 40]. *CFB* is paralogous with complement component 2 (*C2*). These genes are in tandem in the major histocompatibility complex class III region, a cluster of immune-related genes on chromosome 6p and that carry AMD risk [41]. *CFI* is a co-factor along with *CFH* for the inactivation of *C3b*. Noncoding polymorphisms adjacent to the gene and in an intron of CFI are associated with an altered risk of AMD [42]. The complement cascade is a soluble part of the innate immune system. Three different complement pathways activate enzyme cascades that ultimately lead to cell death. Each of the three complement pathways (alternative, lectin, and classical) is a unique method of activating the C3 molecule, initiating pro-inflammatory reactions, and activating the terminal complement pathway. Key to the amplification of the pathway is the fact that C3 is proteolytically activated to C3b due to C3 cleavage and forms a new C3 convertase molecule that will cleave more C3. This result in the production of C3bBb3b in all pathways in addition to C4b2b3b in the classical and lectin pathways, molecules that act as C5 convertases [43]. The convertase cleaves C5 into C5a (an anaphylatoxin) and C5b, which forms part of the membrane attack complex. C5b binds to C6, C7, C8, and C9 in sequence and C8 binds the incomplete membrane attack complex to cell surfaces and up to 16 C9 molecules are then assembled on the membrane to generate a circular polymer. The membrane attack complex can also bind to self-membranes, stimulating the release of growth factors from vascular endothelium. C3a, C4a, and C5a anaphylatoxins are released during these reactions. They are chemoattractants for phagocytic cells [44].

Furthermore, genetic variations (DNA polymorphisms) are known to be associated with phenotypic variation and may alter gene expression patterns [45]. A genetic locus at chromosome 10q26 provides strong susceptibility to AMD; specifically, two AMD-associated polymorphisms near ARMS2 and HTRA1 genes have been suggested to alter the gene expression of either one or the other gene [10]. One study recently reported that haplotypes with del443ins54 and rs11200638 variants influence HTRA1 and ARMS2 expression in genotyped human placentas [46]. However, another report presented that the del443ins54 variant does not change ARMS2 mRNA stability in lymphocytes [11], which is consistent with our data from retina samples. The polymorphic indel at the ARMS2 3′ UTR was associated with AMD in Caucasian and Japanese data sets [47]. This indel is strongly associated with AMD.

There are a number of limitations of current work associated with the available dataset and focus of the current study on chemokine and complement pathways. First, the current analysis includes both retinal and RPE/choroid material datasets that may lead to underrepresentation of retina-specific and RPE/choroid-specific DEG’s output.

Second, only peripheral retinal material was available for the analysis as a limitation of original SRP107937 datasets generation; thus macular transcriptome events were potentially missing from the analysis. Third, human donor data early and late-stage AMD, as well as RPE degeneration samples were included in the analysis with the equal weight, thus potentially increasing heterogenicity. Fourth, retina and RPE/choroid datasets from the same donor were included as individual samples with equal weight. Further analytical efforts that can address some of the above-listed limitations and answer the question regarding tissue-specific events in retina and RPE/choroid are required.

## Conclusions

The transcriptome-wide data analysis provides new insights into the differences in gene regulation between healthy and AMD conditions in the human eye. Analyses of gene ontology, functional pathway analysis, and transcriptional regulation networks of differentially expressed genes showed that chemokine activity, cytokine activity, and immune system activity are dominant during the AMD development, which is driven by the higher expression of the chemokine signaling pathway, complement cascade pathway, and cytokine signaling pathway genes. Among the DEGs, the *CCL3*, *ACKR4*, *CCL19*, *CCL2*, *CXCL10*, *PPBP*, *CXCL9*, *CCL13*, *CCR1*, *CCL21*, *ACKR3*, *CXCL16*, *CCR5*, *CCL4*, and *CXCR6* (chemokine signaling pathway genes); *C5AR1*, *CFH*, *C1R*, *C5AR2*, *CFD*, *C1QB*, *C1QC*, *CD59*, *CFP*, *C3AR1*, *C3*, *CR1*, *C1QA*, and *C1S* (complement cascade pathway genes); and cytokine signaling pathway genes were identified as potential novel regulators of AMD.

## Additional file

Additional file 1: **Figure S1.** Gene network analysis. Chemokine signaling pathway (red color spheres), complement and coagulation cascades pathway (green color spheres) and cytokine-cytokine receptor interactions pathway (blue color spheres). **Figure S2.** A. Extract transcript clusters by expression profile by cutting the dendrogram. B. Principle component analysis, X and Y axis show principle component 1 and principle component 2 that explain 98% and 2% of the variance. **Table S1.** Human normal and AMD of paired-end raw data before and after removal of adapters and retained reads percentages. **Table S2.** List of complement cascade pathway genes, UniProtKB, gene symbol, GenBank id, Gene name, logFC, *p*-value KEGG pathways. **Table S3.** List of Cytokine signaling in immune system pathway genes, UniProtKB, gene symbol, GenBank id, Gene name, logFC, p-value KEGG pathways. (DOCX 920 kb)
