# Supplementary material for: Transcriptome-wide analysis of differentially expressed chemokine receptors, SNPs, and SSRs in the age-related macular degeneration
Source: Hum Genomics. 2019 Mar 20;13:15. doi: 10.1186/s40246-019-0199-1 (PMC6425613; doi:10.1186/s40246-019-0199-1)
Supplement: Supplementary file 1 — Figure S1. Gene network analysis. Chemokine signaling pathway (red color spheres), complement and coagulation cascades pathway (green color spheres) and cytokine-cytokine receptor interactions pathway (blue color spheres). Figure S2. A. Extract transcript clusters by expression profile by cutting the dendrogram. B. Principle component analysis, X and Y axis show principle component 1 and principle component 2 that explain 98% and 2% of the variance. Table S1. Human normal and AMD of paired-end raw data before and after removal of adapters and retained reads percentages. Table S2. List of complement cascade pathway genes, UniProtKB, gene symbol, GenBank id, Gene name, logFC, p-value KEGG pathways. Table S3. List of Cytokine signaling in immune system pathway genes, UniProtKB, gene symbol, GenBank id, Gene name, logFC, p-value KEGG pathways. (DOCX 920 kb) [file 40246_2019_199_MOESM1_ESM.docx]

**Transcriptome-wide Analysis of Differentially Expressed Chemokine Receptors, SNPs and SSRs in the Age-related Macular Degeneration (Supplementary data)**

Madhu Sudhana Saddala^1,2,#^, Anton Lennikov^1,2,#^, Anthony Mukwaya^3^, Lijuan Fan^1,2^, Zhengmao Hu^4^, Hu Huang^1,2^*

*^1^ Mason Eye Institute, University of Missouri, Columbia, MO, 65212, USA*

*^2^Wilmer Eye Institute, Johns Hopkins University, Baltimore, 21287, MD, USA*

*^3^Department of Ophthalmology, Institute for Clinical and Experimental Medicine, Faculty of Health Sciences, Linköping University, Linköping, SE-581 83, Sweden.*

*^4^Center for Medical Genetics & Hunan Key Laboratory of Medical Genetics, School of Life Sciences, Central South University*

^#^Madhu Sudhana Saddala and Anton Lennikov have contributed equally to this work.

*Corresponding author:

Hu Huang, PhD

Department of Ophthalmology

School of Medicine

University of Missouri-Columbia

1 Hospital Drive, MA102C

Columbia, MO 65212

Phone: 573-882-9899

huangh1@missouri.edu


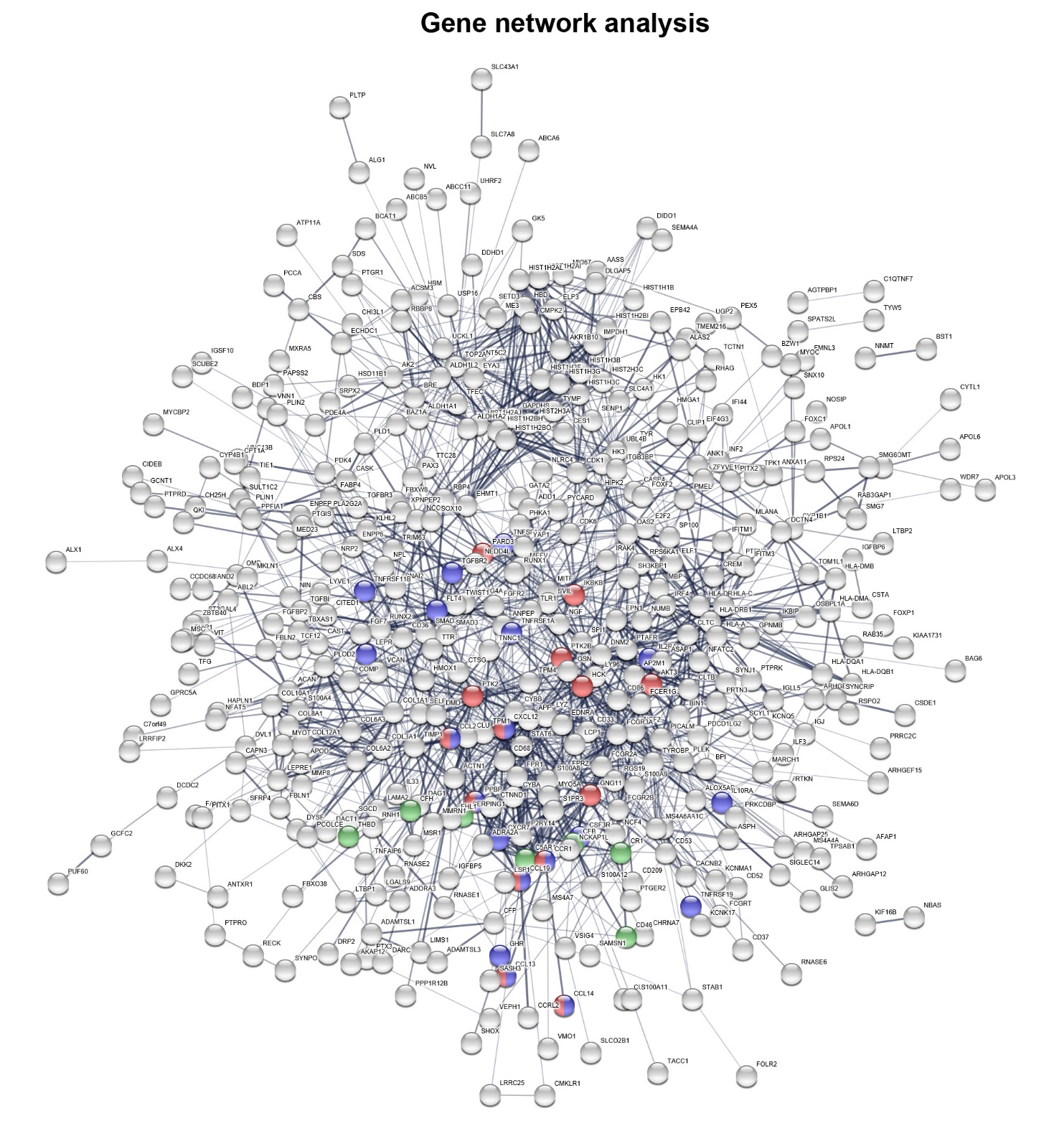


**Additional file 1: Figure S1** Gene network analysis. Chemokine signaling pathway (red color spheres), complement and coagulation cascades pathway (green color spheres) and cytokine-cytokine receptor interactions pathway (blue color spheres).


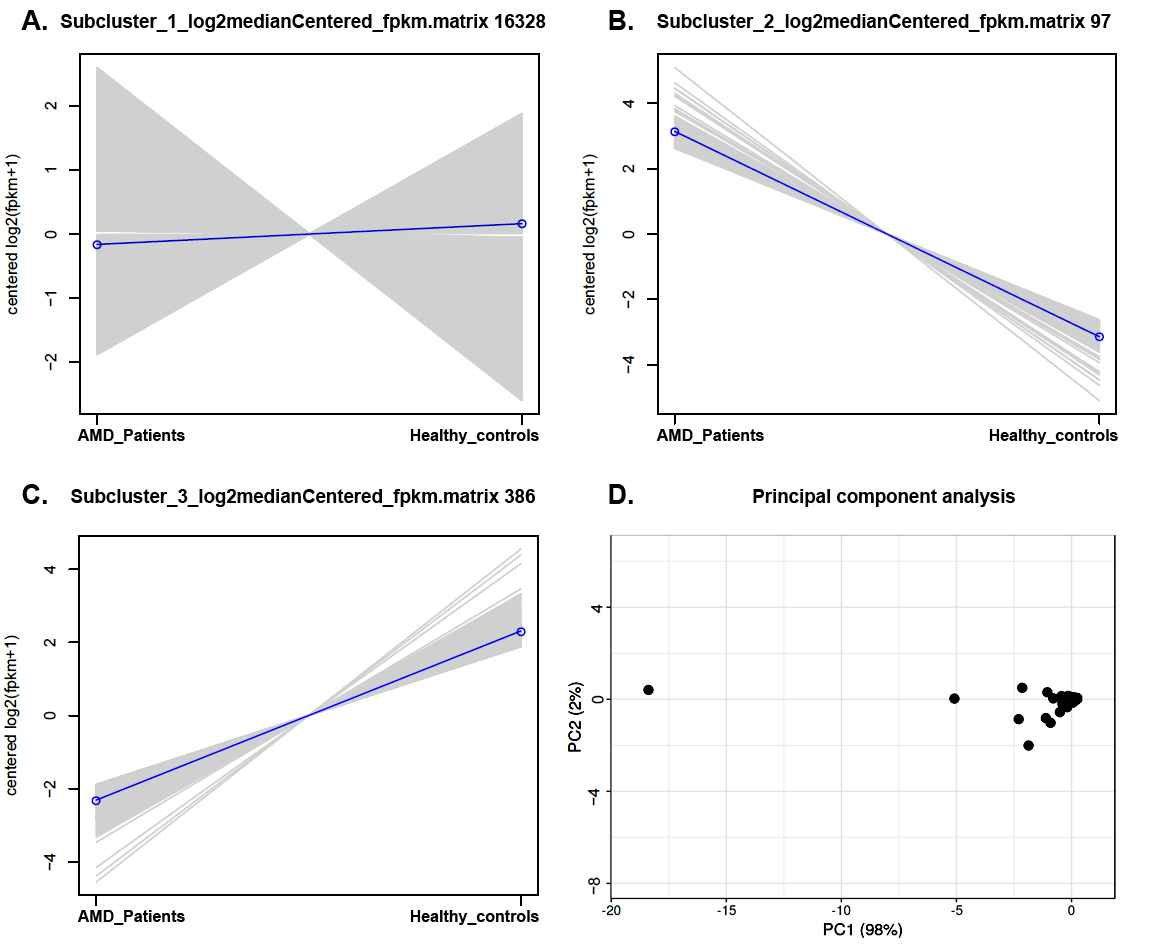


**Figure S2** A. Extract transcript clusters by expression profile by cutting the dendrogram. B. **Principle component analysis**, X and Y axis show principle component 1 and principle component 2 that explain 98% and 2% of the variance.

**Table S1** Human normal and AMD of paired-end raw data before and after removal of adapters and retained reads percentages.

| ***Samples*** | ***Donor Id*** | ***Reads before cleaning*** | ***Reads after cleaning*** | ***Retained reads after trimming (%)*** |
| --- | --- | --- | --- | --- |
| SRR5591599 | Normal_1 | 228356374 | 223274040 | 97.77 |
| SRR5591600 | Normal_1 | 99278204 | 98362985 | 99.07 |
| SRR5591601 | Normal_2 | 93102166 | 92087300 | 98.90 |
| SRR5591602 | Normal_3 | 104389714 | 102914811 | 98.58 |
| SRR5591603 | Normal_3 | 116274614 | 114712047 | 98.65 |
| SRR5591604 | Normal_4 | 84612673 | 83790148 | 99.02 |
| SRR5591605 | Normal_4 | 102123003 | 101020063 | 98.91 |
| SRR5591606 | Normal_5 | 108649007 | 107351335 | 98.80 |
| SRR5591607 | Normal_5 | 101820297 | 100449567 | 98.65 |
| SRR5591608 | Normal_6 | 96166369 | 95115635 | 98.90 |
| SRR5591609 | Normal_6 | 105321973 | 104050698 | 98.79 |
| SRR5591610 | Normal_7 | 154858546 | 153504103 | 99.12 |
| SRR5591611 | Normal_7 | 99056286 | 98090530 | 99.02 |
| SRR5591612 | Normal_8 | 95121437 | 94251563 | 99.08 |
| SRR5591613 | Normal_8 | 124218869 | 122324508 | 98.47 |
| SRR5591614 | AMD_1 | - | - | - |
| SRR5591615 | AMD_1 | 114598704 | 112587115 | 98.24 |
| SRR5591616 | AMD_2 | 75483625 | 74007543 | 98.04 |
| SRR5591617 | AMD_2 | 114959924 | 112444431 | 97.81 |
| SRR5591618 | AMD_3 | 136540007 | 134018559 | 98.15 |
| SRR5591619 | AMD_3 | 118941425 | 116553792 | 97.99 |
| SRR5591620 | AMD_4 | 112212345 | 109909463 | 97.94 |
| SRR5591621 | AMD_4 | 123884462 | 121340560 | 97.94 |
| SRR5591622 | AMD_5 | 112212345 | 98649622 | 87.91 |
| SRR5591623 | AMD_5 | 93858589 | 91781798 | 97.78 |
| SRR5591624 | AMD_6 | 111096635 | 108952279 | 98.06 |
| SRR5591625 | AMD_6 | 116035083 | 113739297 | 98.02 |
| SRR5591626 | AMD_7 | 105703058 | 103947090 | 98.33 |
| SRR5591627 | AMD_7 | 106537923 | 104680087 | 98.25 |
| SRR5591628 | AMD_8 | 142614267 | 140032862 | 98.18 |
| SRR5591629 | AMD_8 | 108432610 | 106507280 | 98.22 |

**Table S2** List of complement cascade pathway genes, UniProtKB, gene symbol, GenBank id, Gene name, logFC, p-value KEGG pathways.

| ***UniProtKB*** | ***Gene Symbol*** | ***GenBank ID*** | ***Gene name*** | ***logFC*** | ***p-value*** | ***KEGG*** |
| --- | --- | --- | --- | --- | --- | --- |
| P21730 | C5AR1 | NM_001736 | C5a anaphylatoxin chemotactic receptor 1 | 5.990090645 | 2.64E-13 | hsa:728 |
| P08603 | CFH | NM_000186 | Complement factor H | 6.015445591 | 1.14E-13 | hsa:3080 |
| P00736 | C1R | NM_001733 | Complement C1r subcomponent | 6.15598173 | 4.56E-14 | hsa:283314 |
| Q9P296 | C5AR2 | NM_001271749 | C5a anaphylatoxin chemotactic receptor 2 | 6.675185538 | 6.30E-11 | hsa:27202 |
| P00746 | CFD | NM_001317335 | Complement factor D | 9.951761555 | 3.45E-07 | hsa:3998 |
| P02746 | C1QB | XM_011542059 | Complement C1q subcomponent subunit B | 6.986619579 | 1.06E-14 | hsa:713 |
| P02747 | C1QC | NM_172369 | Complement C1q subcomponent subunit C | 5.647358147 | 1.48E-12 | hsa:714 |
| P13987 | CD59 | NM_203329 | CD59 glycoprotein | 11.0213876 | 2.24E-10 | hsa:966 |
| P27918 | CFP | NM_001145252 | Properdin | 5.939539147 | 2.32E-12 | hsa:5199 |
| Q16581 | C3AR1 | NM_004054 | C3a anaphylatoxin chemotactic receptor | 4.567947528 | 2.36E-09 | hsa:719 |
| P01024 | C3 | NM_000064 | Complement C3 | 4.701182029 | 6.59E-10 | hsa:711 |
| P17927 | CR1 | NM_000651 | Complement receptor type 1 | 15.10819705 | 1.03E-22 | hsa:2826 |
| P02745 | C1QA | NM_015991 | Complement C1q subcomponent subunit A | 6.969070663 | 2.00E-16 | hsa:712 |
| P09871 | C1S | XM_005253760 | Complement C1s subcomponent | 12.96840596 | 2.87E-16 | hsa:716 |

**Table S3** List of Cytokine signaling in immune system pathway genes, UniProtKB, gene symbol, GenBank id, Gene name, logFC, p-value KEGG pathways.

| ***UniProtKB*** | ***Gene Symbol*** | ***GenBank*** | ***Gene Name*** | ***logFC*** | ***p-value*** | ***KEGG*** |
| --- | --- | --- | --- | --- | --- | --- |
| O43432 | EIF4G3 | NM_001198801 | Eukaryotic translation initiation factor 4 gamma 3 | 11.35684657 | 2.16E-11 | hsa:8672 |
| Q30154 | HLA-DRB5 | NM_002125 | HLA class II histocompatibility antigen, DR beta 5 chain | 10.06443505 | 5.81E-25 | hsa:3127 |
| O00635 | TRIM38 | XM_005248799 | E3 ubiquitin-protein ligase TRIM38 | 11.96858596 | 2.97E-13 | hsa:10475 |
| P01730 | CD4 | NM_000616 | T-cell surface glycoprotein CD4 | 4.249917597 | 1.52E-08 | hsa:3672 |
| P01920 | HLA-DQB1 | NM_002123 | HLA class II histocompatibility antigen, DQ beta 1 chain | 7.59067663 | 3.63E-18 | hsa:3119 |
| P46940 | IQGAP1 | NM_003870 | Ras GTPase-activating-like protein IQGAP1 | 4.361489761 | 6.00E-09 | hsa:8826 |
| Q01628 | IFITM3 | NM_021034 | Interferon-induced transmembrane protein 3 | 5.183145306 | 2.99E-11 | hsa:10410 |
| P13747 | HLA-E | NM_005516 | HLA class I histocompatibility antigen, alpha chain E | 4.160366364 | 2.07E-08 | hsa:3133 |
| Q13478 | IL18R1 | NM_003855 | Interleukin-18 receptor 1 | 5.525442688 | 3.43E-11 | hsa:8809 |
| P10398 | ARAF | NM_001256196 | Serine/threonine-protein kinase A-Raf | 10.02208151 | 1.94E-07 | hsa:51669 |
| P02751 | FN1 | NM_212476 | Fibronectin | 4.705209929 | 7.46E-10 | hsa:22885 |
| P01903 | HLA-DRA | NM_019111 | HLA class II histocompatibility antigen, DR alpha chain | 4.644297721 | 9.50E-10 | hsa:3122 |
| P01589 | IL2RA | NM_001308242 | Interleukin-2 receptor subunit alpha | 4.789258721 | 1.87E-06 | hsa:3559 |
| P01911 | HLA-DRB1 | NM_002124 | HLA class II histocompatibility antigen, DRB1-15 beta chain | 6.36794337 | 1.11E-14 | hsa:105369230 |
| P32456 | GBP2 | NM_004120 | Guanylate-binding protein 2 | 6.259017091 | 2.81E-14 | hsa:10659 |
| P40305 | IFI27 | NM_001288956 | Interferon alpha-inducible protein 27, mitochondrial | 13.23585581 | 4.51E-17 | hsa:122509 |
| P14778 | IL1R1 | XM_005263934 | Interleukin-1 receptor type 1 | 12.58280743 | 4.17E-15 | hsa:3554 |
| O00300 | TNFRSF11B | NM_002546 | Tumor necrosis factor receptor superfamily member 11B | 6.261318784 | 1.48E-12 | hsa:690 |
| P13284 | IFI30 | NM_006332 | Gamma-interferon-inducible lysosomal thiol reductase | 7.348063093 | 2.31E-17 | hsa:10437 |
| P13164 | IFITM1 | NM_003641 | Interferon-induced transmembrane protein 1 | 5.863265658 | 3.47E-13 | hsa:8519 |
| Q14627 | IL13RA2 | NM_000640 | Interleukin-13 receptor subunit alpha-2 | 4.545729473 | 1.24E-06 | hsa:3598 |
| O15520 | FGF10 | NM_004465 | Fibroblast growth factor 10 | 5.775407954 | 5.26E-09 | hsa:101927075 |
| P61769 | B2M | NM_004048 | Beta-2-microglobulin | 4.867621658 | 2.23E-10 | hsa:64216 |
| P13725 | OSM | NM_020530 | Oncostatin-M | 5.595401143 | 1.16E-09 | hsa:83605 |
| Q02763 | TEK | NM_000459 | Angiopoietin-1 receptor | 4.121276734 | 3.68E-08 | hsa:100233156 |
| O15524 | SOCS1 | NM_003745 | Suppressor of cytokine signaling 1 | 4.102733564 | 8.36E-06 | hsa:8651 |
| P19438 | TNFRSF1A | NM_001346092 | Tumor necrosis factor receptor superfamily member 1A | 4.702363928 | 2.71E-09 | hsa:8717 |
| Q8N2H9 | PELI3 | NM_001243136 | E3 ubiquitin-protein ligase pellino homolog 3 | 10.98663911 | 2.78E-10 | hsa:246330 |
| P05362 | ICAM1 | NM_000201 | Intercellular adhesion molecule 1 | 4.799471897 | 4.17E-10 | hsa:148022 |
| Q6AZZ1 | TRIM68 | NM_001304496 | E3 ubiquitin-protein ligase TRIM68 | 10.98663911 | 2.78E-10 | hsa:55128 |
| P24158 | PRTN3 | NM_002777 | Myeloblastin | 6.53239753 | 1.83E-11 | hsa:5657 |
| Q01629 | IFITM2 | NM_006435 | Interferon-induced transmembrane protein 2 | 4.438385337 | 4.17E-09 | hsa:10581 |
| P25105 | PTAFR | NM_001164723 | Platelet-activating factor receptor | 10.38434323 | 1.83E-08 | hsa:5724 |
| O95760 | IL33 | NM_001314046 | Interleukin-33 | 6.486004689 | 6.12E-14 | hsa:90865 |
| Q8WU20 | FRS2 | XM_017018718 | Fibroblast growth factor receptor substrate 2 | 11.85758345 | 6.37E-13 | hsa:6427 |
| O14508 | SOCS2 | NM_001270467 | Suppressor of cytokine signaling 2 | 4.358550065 | 6.43E-08 | hsa:8835 |
| P55265 | ADAR | NM_015841 | Double-stranded RNA-specific adenosine deaminase | 4.344109939 | 1.20E-08 | hsa:103 |
| P20036 | HLA-DPA1 | NM_033554 | HLA class II histocompatibility antigen, DP alpha 1 chain | 4.153393324 | 2.16E-08 | hsa:3113 |
| P26441 | CNTF | NM_000614 | Ciliary neurotrophic factor | 10.21455314 | 5.22E-08 | hsa:1271 |
| P01909 | HLA-DQA1 | NM_002122 | HLA class II histocompatibility antigen, DQ alpha 1 chain | 5.392061304 | 9.22E-12 | hsa:3117 |
| Q02556 | IRF8 | NM_002163 | Interferon regulatory factor 8 | 4.50406715 | 5.37E-09 | hsa:3394 |
| P16871 | IL7R | NM_002185 | Interleukin-7 receptor subunit alpha | 12.13649104 | 9.17E-14 | hsa:3575 |
| P42229 | STAT5A | NM_001288720 | Signal transducer and activator of transcription 5A | 10.48736228 | 9.62E-09 | hsa:6776 |
